# Observation of quantum recoherence of photons by spatial propagation

**DOI:** 10.1038/srep15330

**Published:** 2015-10-15

**Authors:** Frédéric Bouchard, Jérémie Harris, Harjaspreet Mand, Nicolas Bent, Enrico Santamato, Robert W. Boyd, Ebrahim Karimi

**Affiliations:** 1 Department of Physics, University of Ottawa, 25 Templeton St., Ottawa, Ontario, K1N 6N5 Canada; 2 Dipartimento di Scienze Fisiche, Universitá di Napoli “Federico II”, Complesso di Monte S. Angelo, 80126 Napoli, Italy; 3 Institute of Optics, University of Rochester, Rochester, New York, 14627, USA

## Abstract

Entanglement is at the heart of many unusual and counterintuitive features of quantum mechanics. Once two quantum subsystems have become entangled, it is no longer possible to ascribe an independent state to either; instead, the subsystems are completely described only as part of a greater, composite system. As a consequence of this, each entangled subsystem experiences a loss of coherence following entanglement. We refer to this decrease in coherence as *decoherence*. Decoherence leads inevitably to the leaking of information from each subsystem to the composite entangled system. Here, we demonstrate a process of decoherence reversal, whereby we recover information lost from the entanglement of the optical orbital angular momentum and radial profile degrees of freedom possessed by a photon pair. These results carry great potential significance, since quantum memories and quantum communication schemes depend on an experimenter’s ability to retain the coherent properties of a particular quantum system.

When one quantum system becomes entangled with another, its purity is diminished as phase information is lost to the second (ancilla) Hilbert space. Once the system has lost coherence, its state cannot be described independently of the ancilla. The system is said to have undergone decoherence, and the combined state of the system and ancilla must be specified in order to capture the information originally contained in the system alone^1^,^2^.

In quantum computation and communication protocols, only the state of the system is generally accessible, meaning that its entanglement with an ancilla produces a loss of available information^3^. Therefore, the ability to reverse the decoherence process and recover the original system state holds great promise in many areas^4^,^5^,^6^,^7^. We demonstrate the reversal of propagation-induced decoherence, which we term *recoherence*. We show that quantum information in the orbital angular momentum (OAM) degree of freedom of an entangled photon pair can be lost and retrieved through propagation, by manipulating the degree of entanglement (DOE) between their OAM and radial mode Hilbert spaces. This effect should not be confused with entanglement migration, in which information is transferred between wavefunction phase and amplitude by propagation, rather than having been lost to ancilliary Hilbert spaces^8^, nor should it be mistaken for quantum erasure, which is achieved by information loss due to projective measurement^9^. The effects explored here are of more general interest, as they are brought upon by the action of a unitary operation (free spatial propagation) that is usually assumed to preserve OAM information content. The general decoherence/recoherence paradigm is illustrated in Fig. 1, which shows an initial state consisting of a system 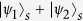 and ancilla 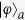, which are entangled by the decoherence process, producing a combined state 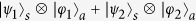. This entanglement is then reversed by (unitary) spatial propagation, and the original state recovered.

We observed the recoherence effect by choosing as the system the OAM degrees of freedom of an entangled photon pair, and by taking the photons’ radial profiles to represent the ancilla. The entangled pair was generated from a pump photon produced in a Laguerre-Gauss (LG) mode. LG modes are a well-characterized family of solutions to the paraxial wave equation, and form an orthonormal and complete basis^10^. LG beams are of particular interest since they represent eigenstates of OAM. They carry an OAM of 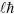 per photon^11^ and are given by


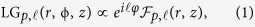


where *p* and 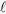 are the modal radial and azimuthal indices, and *r*, *ϕ*, *z* are the standard cylindrical coordinates. In this representation, the dependence of the LG transverse profile on the radial and longitudinal coordinates *r* and *z* is entirely contained within 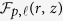
12, which possesses a doughnut-shaped amplitude for 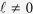, and a Gaussian radial profile for 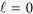.

## Results

Recently, optical OAM conservation has been demonstrated during spontaneous parametric down conversion (SPDC)^13^,^14^. In SPDC, a single photon is frequency down-converted in a nonlinear material to produce two photons whose total OAM is equal to that of the incident pump photon. The generated photons are known as the signal and idler, and will in general belong to modes with different OAM values. Consequently, their radial modes will evolve differently upon free-space propagation. Notwithstanding the disparate modal evolution of the signal and idler, the transverse profiles of both SPDC photons must overlap within the nonlinear crystal immediately following down conversion, to ensure continuity of field intensities during SPDC.

We consider the case *p* = 0 and take 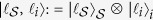 to denote an optical state in which the signal (idler) photon carries 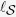 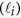 units of OAM, and 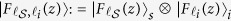 to represent the radial profiles 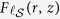 and 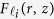 of the signal and idler, where we have dropped the index *p* for simplicity, and the subscripts *s* and *i* indicate the signal and idler photons, respectively. For an incident pump photon with 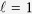, the state of the SPDC pair is


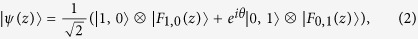


where *θ* is the phase between the SPDC photon pair states. Here we account only for signal and idler photons generated with either 0 or 1 units of OAM (see Supplementary Information (SI)).

Immediately following SPDC, continuity of the optical fields in the nonlinear crystal requires that 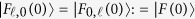. Consequently, the radial profile component of the total state can be factored, and 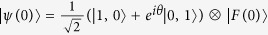 (see SI). Therefore, the OAM state of the system is pure immediately following photon pair generation, and the radial and OAM state components of 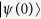 are separable. Upon propagation, this separability disappears as the transverse profiles become entangled with the OAM state of each photon, so that for *z* ≠ 0, the two-photon state is described by Eq. (2) instead, and cannot be factorized. The unitary operator responsible for the entanglement of the OAM and radial mode Hilbert spaces is 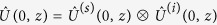, with 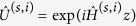, 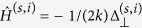 and where 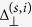 denotes the transverse laplacian over the signal and idler Hilbert spaces, respectively. In the position representation, 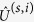 takes the form of the Fresnel propagator (see SI).

The propagation effects described can be understood by considering the reduced density matrix 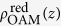 of the OAM Hilbert space alone. This matrix is obtained by tracing over the radial profile component of the total density matrix 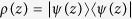, and is found to take the form 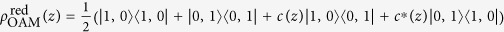, where 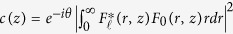 (see SI). The reduced density matrix can be expressed alternatively as


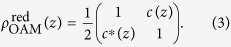


The off-diagonal terms take values *c*(0) = exp (−*iθ*) in the SPDC plane, and their magnitudes decrease with propagation distance *z*. This decreases the purity of the reduced density matrix, and increases the entanglement entropy of the OAM subspace. The purity of the reduced density matrix defined above is given by 
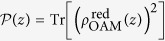
, where Tr[⋅] denotes the trace operation. The entanglement entropy of the OAM subspace is 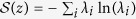, where *λ*_*i*_ are the eigenvalues of 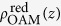, and ln (⋅) denotes the natural logarithm. Purity and entanglement entropy represent measures of the DOE of the system and ancilla; as 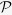 (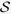) decreases (increases), the system and ancilla become increasingly entangled (and *vice-versa*). As a point of physical interest, we note that the decoherence of the OAM Hilbert space of the state presented in Eq. (2) does not occur due to the introduction of *ad hoc* classical noise in the phase term; rather, it arises from ignorance regarding ancillary degree (s) of freedom (radial mode) entangled with the quantum system under study (OAM).

For the sake of clarity, we have included the radial degree of freedom of the photon pairs in our analysis from the very beginning. Decoherence and recoherence effects due to the non-trivial evolution of the radial degree of freedom, are then derived straightforwardly. This treatment may lead the reader to think this effect unsurprising or rather obvious. Nevertheless, the quantum nature of the photonic radial degree of freedom has only recently been recognized and demonstrated^15^. Hence a naïve analysis of the propagation of OAM-entangled photon pairs would assume photon OAM to be robust against spatial propagation. Indeed, this error would be all the more understandable given that its underlying assumptions hold true for single photons; it is the fact that a multi-photon system is considered here that makes decoherence an issue. Unfortunately, quantum key distribution and quantum information applications depend heavily upon precisely the sort of multi-photon entanglement discussed here^16^. We note also that this coherence retrieval process is physically distinct from non-unitary projective post-selection, which can achieve similar ends, but entails the loss of a substantial number of photons. Moreover, the recoherence of a quantum photonic state arising from purely unitary evolution in space (or equivalently in time), is a completely novel, and hitherto unobserved, effect.

**Figure 2:**
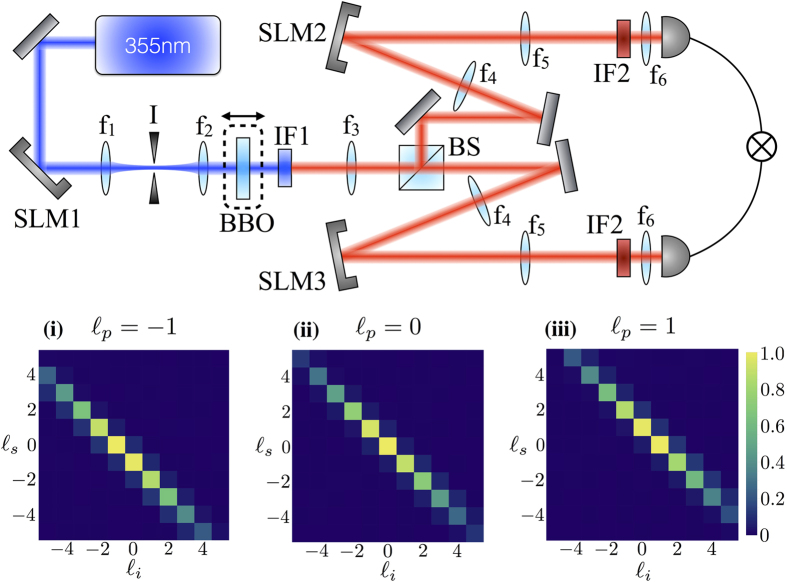
Experimental setup. Using SLM1, we impart an OAM of 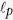 per photon on a beam of UV light, which is made to pass through a Type I *β*-barium borate (BBO) crystal for SPDC. The entangled photon pair is separated by a beam splitter, and each photon is imaged on a different SLM. Only when the pattern displayed on the SLM is complementary to the phase front of the incident photon will it be phase-flattened and coupled to a single-mode optical fibre for eventual detection by a coincidence counter. The BBO crystal is mounted on a translational stage, so that SPDC fields imaged on the SLMs correspond to different propagation ranges. Insets **(i)**, **(ii)** and **(iii)** show experimental OAM conservation matrices obtained for pump OAM values of 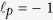, 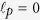 and 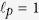. These results confirm that OAM conservation is indeed verified in our optical system, indicating that the setup is capable of reliably measuring photon OAM.

Our experimental setup is shown in Fig. 2. The output of a quasi-continuous wave UV laser operating with a repetition rate of 100 MHz and an average power of 150 mW at a wavelength of 355 nm is spatially cleaned and sent to a kinoform displayed on a Hamamatsu UV spatial light modulator (SLM1), which reshapes the spatial distribution of the incoming beam into a desired 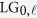 mode. The first diffracted order obtained from the reflection of the beam off the SLM is selected using an iris placed at the Fourier plane of the lens *f*_1_ = 200 mm. The beam is then imaged from the UV SLM to a *L* = 3 mm thick type-I *β*-barium borate (BBO) crystal by means of a 4*f*-system (*f*_1_ = 200 mm – *f*_2_ = 100 mm). The BBO crystal is placed on a translational stage so as to allow the SPDC output to be imaged and analyzed for different propagation distances. The SPDC photons are separated into two different paths at a non-polarising beam splitter and then re-imaged on SLMs 2 and 3 (Hamamatsu) by means of additional 4*f*-systems (*f*_3_ = 400 mm – *f*_4_ = 200 mm). SLM 2 and SLM 3 perform the measurement on the OAM state of the down-converted photons. The measured photons are then coupled to single mode optical fibres and sent to avalanche photodiode (APD) detectors. The coincidence counts between the two detectors are measured by means of a coincidence box with a time window of 10 ns.

We generate OAM-entangled photon pairs from SPDC, which at the BBO crystal have identical radial intensity profiles. Upon propagation, their radial profiles will differ increasingly, causing system decoherence, and a decrease (increase) in the purity (entropy) of the photons’ OAM states. By imaging the exit plane of the BBO crystal on a SLM and single mode optical fibre, we can retrieve the initial radial profiles, and achieve recoherence by recovering maximal OAM state purity. The BBO crystal is then mounted on a translational stage and moved along the beam axis to image different propagation planes, where only partial recoherence occurs. The purity of the OAM state is calculated from reconstructed density matrices obtained from OAM state tomography performed over the Hilbert space spanned by the basis 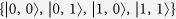. Tomography is carried out by using SLMs and single mode optical fibres, which in combination carry out a function analogous to that of the polariser in polarisation state tomography^17^,^18^. Our apparatus allows the DOE between the OAM and radial profile states to be adjusted and measured with precision.

**Figure 3:**
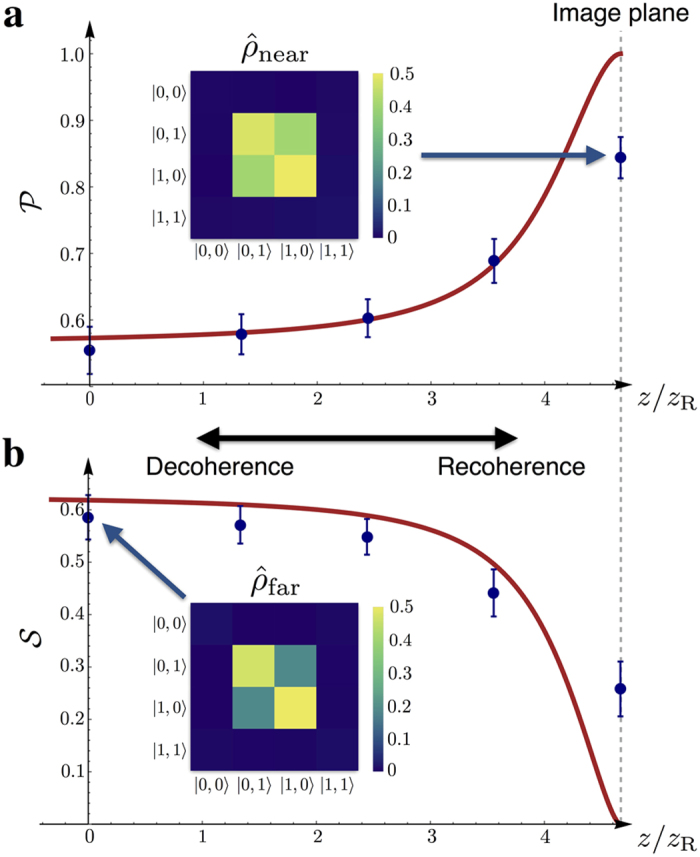
Purity and von Neumann entropy of the OAM state for different SPDC propagation distances. (**a**) Experimental and theoretical plots of OAM state purity. Experimental data were obtained over a propagation range of 4.7*z*_R_ = 21 mm (see text), and the theoretical plot from the reconstructed density matrix Eq. (3) obtained from propagation simulations described in the main text and SI. (**b**) Experimental and theoretical plots of OAM state von Neumann entropy. Experimental data correspond to those plotted in part (**a**). Insets show reconstructed density matrices 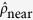 and 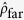 obtained from experimental data at *z* = 0 and *z* = 21 mm (the near- and far-field positions). Only the absolute values of the matrices’ off-diagonal elements were plotted, since their phases do not influence system purity or entropy. As can be seen from the insets, the magnitude of the off-diagonal elements visibly increases as the BBO crystal is translated toward the detection image plane, accounting for the corresponding increase in system purity.

Figure 3a shows plots of system purity versus imaging position. The theoretically predicted increase of purity with imaging position is verified experimentally. Neither the theoretical nor the experimental curves reach their minimum possible purity of *P* = 1/2; hence, the OAM and radial profile Hilbert spaces are never maximally entangled (see SI). Instead, measured OAM state purities ranged from 0.55 to 0.84. In Fig. 3b, we show corresponding plots of system von Neumann entropy. Measured and theoretical values agree qualitatively, decreasing with imaging position, respectively ranging from 0.26 to 0.59, and 0 to 0.62. The purity and von Neumann entropy were found to differ from their theoretical counterparts at the image plane. We attribute this disagreement to the finite thickness of the BBO crystal, which means that in practice, SPDC does not occur in an infinitely thin plane, but within a range (3 mm) of positions in the crystal.

## Discussion

This experiment shows that propagation-induced decoherence can be reversed to recover lost information, provided that a judicious choice of imaging optics is made by the experimenter. Following SPDC, the radial profiles of the signal and idler begin to differ upon propagation. The resulting entanglement between the signal and idler’s respective OAM content and their radial profiles leads to a loss of accessible information from the bi-photon OAM Hilbert space. In our experiment, this information loss was reversed by the action of a lens and free propagation of the SPDC pair. Though the reversal of any quantum mechanical process must always be theoretically possible, these findings demonstrate that this reversibility can be observed in practice, even for decoherence phenomena. Apart from their being of great interest from a fundamental standpoint, our results may suggest a possible means by which to avoid the information loss that can accompany spatial propagation of coherent optical signals. Our findings are relevant to any quantum optical communication or information storage scheme in which information loss from propagation-induced decoherence is a concern.

## Additional Information

**How to cite this article**: Bouchard, F. *et al.* Observation of quantum recoherence of photons by spatial propagation. *Sci. Rep.* **5**, 15330; doi: 10.1038/srep15330 (2015).

## Supplementary Material

Supplementary Information

## Figures and Tables

**Figure 1:**
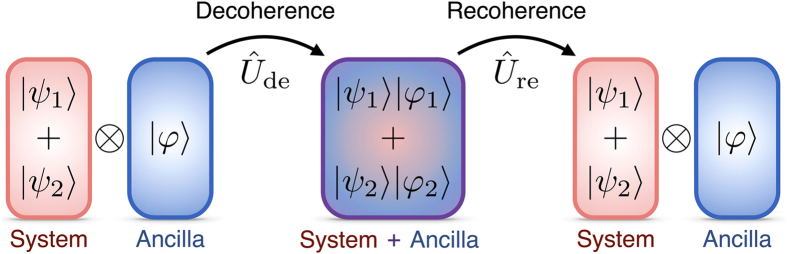
Illustration of the processes of decoherence and recoherence. The system’s initial state ray takes the form 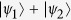, where 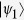 and 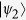 are themselves state rays in the system Hilbert space. Before decoherence, the system and ancilla Hilbert spaces are separable, and a state may be ascribed to each independently. Both kets describing the system are associated with the same ancilla ket 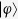. Upon decoherence induced by the unitary operator 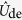, the state of the ancilla becomes contingent on the state of the system, so that the ancilla kets 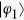 and 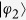, respectively associated with the 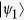 and 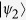 system states, are no longer identical, i.e. 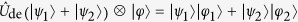. Therefore, the two Hilbert spaces become non-separable, and coherence is lost, if one can only access the system. During the recoherence process, application of a unitary operator 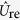 causes the kets 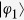 and 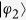 to recover their initial form 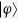, allowing system Hilbert space coherence to be regained, i.e. 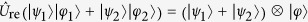.
