# Supplementary material for: Observation of quantum recoherence of photons by spatial propagation
Source: Sci Rep. 2015 Oct 15;5:15330. doi: 10.1038/srep15330 (PMC4606782; doi:10.1038/srep15330)
Supplement: Supplementary Information [file srep15330-s1.pdf]

Supplementary Information for Observation of quantum recoherence of photons by spatial propagation

Frédéric Bouchard,[1] Jérémie Harris,[1] Harjaspreet Mand,[1] Nicolas Bent,[1] Enrico Santamato,[2] Robert W. Boyd,[1,3] and Ebrahim Karimi[1,*]

[1]Department of Physics, University of Ottawa, 25 Templeton St., Ottawa, Ontario, K1N 6N5 Canada

[2]Dipartimento di Scienze Fisiche, Universitá di Napoli "Federico II", Complesso di Monte S. Angelo, 80126 Napoli, Italy

[3]Institute of Optics, University of Rochester, Rochester, New York, 14627, USA

I. OAM CONSERVATION

A parametric process is one in which light interacts with a material in such a way as to leave the quantum state of this material unchanged after the interaction. The initial and final optical states then carry the same energy, linear momentum and angular momentum. The energy conservation condition is fulfilled by requiring the SPDC photon pairs to be frequency down-converted such that the sum of the frequencies of the SPDC photons is equal to the frequency of the pump photon. The down-converted photon pair is then in a frequency entangled state. Similarly, OAM conservation is enforced by the requirement that the SPDC photons be entangled in OAM, so that their state is given by the expression

$$\left|\psi_{\ell_p}\right\rangle = \sum_{m=-\infty}^{\infty} c_m \left|m+\ell_p\right\rangle_s \left|-m\right\rangle_i . \tag{1}$$

As can be seen from Fig. 1, the state $\left|\psi_{\ell_p}\right\rangle$ possess an *OAM spectrum*, $|c_m|^2$. The OAM spectrum can be tuned by carefully adjusting the SPDC phase matching condition of the nonlinear crystal. In the case of a BBO crystal, this can be easily achieved by tilting the crystal. For the case of $\ell_p = 0$, the OAM spectrum peaks at $\ell_{s,(i)} = \ell_{i,(s)} = 0$. As we break the symmetry by pumping the crystal with a photon $\ell_p \neq 0$, the OAM spectrum will peak at $\ell_{s,(i)} = \ell_p$ and $\ell_{i,(s)} = 0$. In this work, we limit our analysis to an OAM entangled state given by: $\left|\psi_{\ell_p}\right\rangle = \frac{1}{\sqrt{2}}\left(\left|\ell_p\right\rangle_s \left|0\right\rangle_i + e^{i\theta}\left|0\right\rangle_s \left|\ell_p\right\rangle_i\right)$.

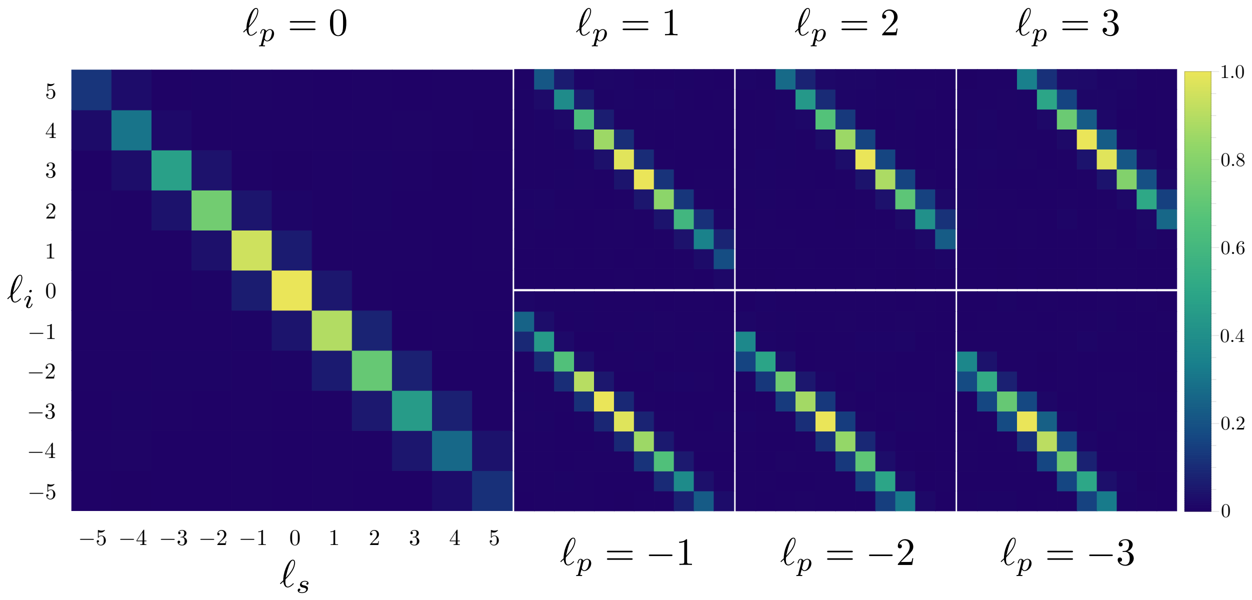

Figure 1: **OAM conservation matrices.** Results obtained from OAM conservation experiments are shown for incident pump beam OAM values of $\ell = -3, -2, -1, 0, 1, 2, 3$. These data show the expected linear relationship between signal and idler OAM values, the sum of the signal and idler OAM indices being exactly equivalent to the pump OAM, verifying angular momentum conservation.

II. SPDC PROPAGATION

If the down-converted (pump) photon incident on the nonlinear crystal is described by OAM and radial indices ℓ and $p = 0$ respectively, it must belong to a mode possessing the corresponding doughnut-shaped intensity pattern $F_\ell^{\text{pump}}(r, z)$. Hence, it will give rise to two SPDC photons for which the transverse modal amplitudes will initially (that is, at $z = 0$) be identical to $F_\ell^{\text{pump}}(r, 0)$. Conservation of OAM also requires that the total OAM of the signal-idler pair be equal to ℓ. One can show (See SI. I) that the most likely distribution of OAM between these photons would find the signal (idler) carrying ℓ units of OAM, while the idler (signal) carries none. As a result, at the SPDC plane, the transverse amplitudes of the generated photon pair will be given by $F_\ell^{s,i}(r, 0)$ and $F_0^{i,s}(r, 0)$, where we must have

$$F_\ell^{\text{pump}}(r, 0) = F_\ell^{s,i}(r, 0) = F_0^{i,s}(r, 0). \tag{2}$$

Upon propagation, however, the transverse amplitudes of the signal and idler modes will become dependent on the photons' respective OAM content. The particular transverse amplitude associated with each mode can be determined at a distance z from the SPDC plane by applying

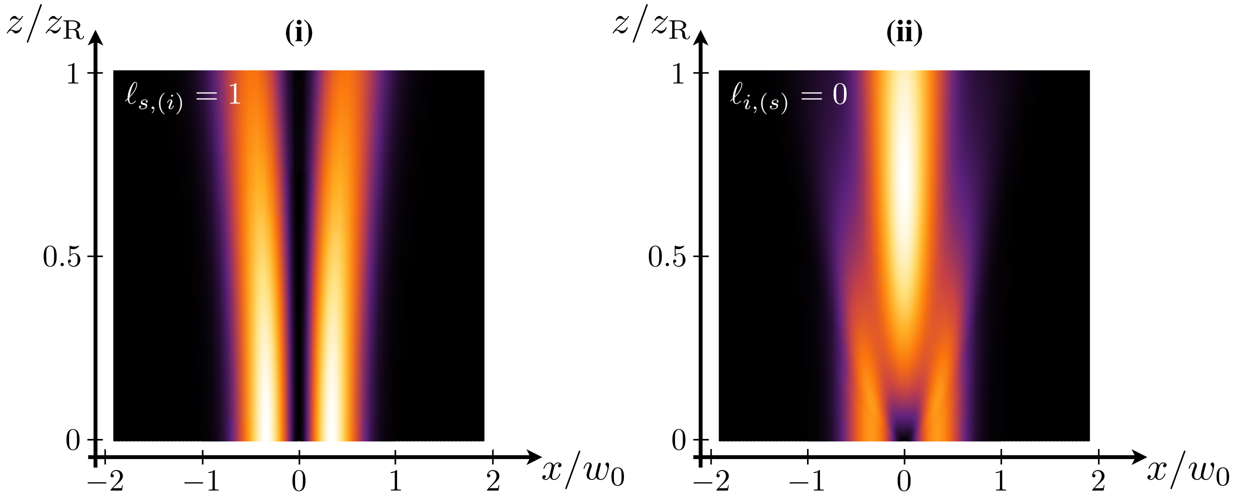

Figure 2: **Propagation simulation.** The intensities of the fields associated with the signal and idler photons generated from SPDC with a pump OAM of $\ell_p = 1$ are shown as a function of propagation distance z and transverse coordinate x, where $r = \sqrt{x^2 + y^2}$. The longitudinal and transverse figure axes are respectively normalised to the Rayleigh range z_R and beam waist w_0. The SPDC process produces a signal (idler) photon with an OAM of $\ell_{s,(i)} = 1$ and an idler (signal) photon with an OAM of $\ell_{s,(i)} = 0$. We show normalised beam intensities for a propagation range of $z/z_R = 0$ to $z/z_R = 1$. Of particular note, the radial intensities for both the signal and idler are found to agree perfectly in the $z = 0$ plane, differing appreciably within from one another within one Rayleigh lenght.

the Fresnel propagator to the signal and idler amplitude functions at $z = 0$, as follows:

$$F_\ell^{s,i}(r, z) = \frac{-i\pi}{\lambda z} e^{\frac{ikr^2}{2z}} \int_0^\infty r'dr' e^{\frac{ikr'^2}{2z}} J_\ell\left(\frac{krr'}{z}\right) F_\ell^{s,i}(r', 0). \tag{3}$$

In the case of a pump photon belonging to a LG mode possessing an OAM index of $\ell = 1$, we have $F_1^{s,i}(r, 0) = F_0^{i,s}(r, 0) = -2i\sqrt{2}\left(\frac{r}{w_0}\right) e^{-\frac{r^2}{w_0^2}}$. In Fig 2, we show corresponding theoretical plots depicting the spatial evolution of the radial SPDC profiles for the case in which the signal photon carries $\ell = 1$ unit of OAM, while the idler carries none. The overlap between these fields decreases rapidly upon propagation, even prior to the Rayleigh range, as can be gathered from the figure. This diminished overlap results in a reduction in the values of the off-diagonal elements $c(z)$ of the reduced density matrix $\rho_{\text{OAM}}^{\text{red}}$, and a corresponding increase in the entanglement entropy of the OAM subsystem upon propagation.

III. THEORETICAL DETERMINATION OF THE OFF-DIAGONAL ELEMENTS OF THE REDUCED DENSITY MATRIX

The full density matrix describing the system (OAM) and ancilla (transverse profile) Hilbert spaces at any arbitrary propagation distance z is given by

$$\begin{aligned}\rho(z) = \frac{1}{2}\Big(& \ |1,0\rangle \otimes |F_{1,0}(z)\rangle \langle 1,0| \otimes \langle F_{1,0}(z)| \\ + & \ |0,1\rangle \otimes |F_{0,1}(z)\rangle \langle 0,1| \otimes \langle F_{0,1}(z)| \\ + & \ e^{-i\theta} |1,0\rangle \otimes |F_{1,0}(z)\rangle \langle 0,1| \otimes \langle F_{0,1}(z)| \\ + & \ e^{i\theta} |0,1\rangle \otimes |F_{0,1}(z)\rangle \langle 1,0| \otimes \langle F_{1,0}(z)| \Big).\end{aligned} \tag{4}$$

The reduced density matrix over the OAM Hilbert space, $\rho_{\mathrm{OAM}}^{\mathrm{red}}$, is obtained by tracing $\rho(z)$ over the ancilla space. This can be achieved by taking

$$\begin{aligned}\rho_{\mathrm{OAM}}^{\mathrm{red}} &= \mathrm{Tr}_a\left[\rho(z)\right] = \\ & \int r_1\, dr_1 \int r_2\, dr_2\, \left(\langle r_2|_i \langle r_1|_s \rho(z) |r_1\rangle_s |r_2\rangle_i\right),\end{aligned} \tag{5}$$

where, for example, $\langle r_2|_i \langle r_1|_s \cdot |F_{1,0}(z)\rangle = F_1(r_1,z)F_0(r_2,z)$. The reduced density matrix then becomes

$$\begin{aligned}\rho_{\mathrm{OAM}}^{\mathrm{red}} = \frac{1}{2}\Big(& \ a(z)\ |1,0\rangle\langle 1,0| + b(z)\,|0,1\rangle\langle 0,1| \\ + & \ c(z)\,|1,0\rangle\langle 0,1| + d(z)\,|0,1\rangle\langle 1,0| \Big),\end{aligned} \tag{6}$$

where we have defined

$$a(z) := \int_0^\infty |F_1(r_1,z)|^2\, r_1 dr_1 \int_0^\infty |F_0(r_2,z)|^2\, r_2 dr_2, \tag{7}$$

$$b(z) := \int_0^\infty |F_0(r_1,z)|^2\, r_1 dr_1 \int_0^\infty |F_1(r_2,z)|^2\, r_2 dr_2, \tag{8}$$

$$c(z) := e^{-i\theta} \int_0^\infty F_1(r_1,z)F_0^*(r_1,z) r_1 dr_1 \int_0^\infty F_1^*(r_2,z)F_0(r_2,z) r_2 dr_2, \tag{9}$$

and

$$d(z) := e^{i\theta} \int_0^\infty F_0(r_1, z) F_1^*(r_1, z) r_1 dr_1 \int_0^\infty F_0^*(r_2, z) F_1(r_2, z) r_2 dr_2. \tag{10}$$

We note immediately that $a(z) = b(z) = 1$ in order to satisfy normalization of the transverse modes, and that $c(z) = d^*(z) = e^{-i\theta} \left| \int_0^\infty F_1^*(r, z) F_0(r, z) r dr \right|^2$. The matrix representation of $\rho_{\mathrm{OAM}}^{\mathrm{red}}$ is therefore given by

$$\rho_{\mathrm{OAM}}^{\mathrm{red}}(z) = \frac{1}{2} \begin{pmatrix} 1 & c(z) \\ c^*(z) & 1 \end{pmatrix}. \tag{11}$$

IV. TOMOGRAPHY

In this section we describe the tomography process used to reconstruct the state of the down-converted photons for different propagation distances. A measurement is made on the OAM state of the photons by means of two SLMs. As described in SI. I, the most probable output OAM state from SPDC pumped by a photon carrying ℓ_p units of OAM is given by: $|\psi\rangle = \frac{1}{\sqrt{2}} \left(|0, \ell_p\rangle + e^{i\theta} |\ell_p, 0\rangle \right)$. Thus, we limit our analysis to a four-dimensional OAM Hilbert space spanned by the following basis: $\{ |0,0\rangle, |0,\ell_p\rangle, |\ell_p, 0\rangle, |\ell_p, \ell_p\rangle \}$. This specific OAM subspace is isomorphous to the two-photon polarisation Hilbert space, which can be represented by the SU(4) group. Tensor products of Pauli matrices $\hat{\sigma}_x$, $\hat{\sigma}_y$, $\hat{\sigma}_z$ and the identity matrix ˆ are generators of the SU(4) group. Thus, one can reconstruct the OAM density matrix of the down-converted photon pairs by projecting the unknown bipartite OAM state over the eigenstates of the generators. This is analogous to measuring the polarisation Stokes parameters in the case of photon pairs. The correspondence with the case of polarisation is straightforwardly achieved by associating $|0\rangle$ and $|\ell_p\rangle$ with the left and right-handed circular polarised states of light, respectively. A full characterisation of the unknown OAM state can be effected by measuring sixteen independent Stokes-like parameters $r_{i,j}$ $(i, j = 0, 1, 2, 3)$. In order to perform these sets of measurements, we implement a well-known phase-flattening projective measurement. In this technique, both photons are imaged onto SLMs where different computer-generated holograms are displayed. These holograms flatten the phase-front of the beam at the first order of diffraction. The flattened component of the optical field can be coupled into a single mode optical fibre. The SLM and the single mode optical fibre act as the polariser in the case of polarisation state tomography. The reconstructed OAM density

matrix of the entangled photon pairs is then obtained from the following expression:

$$\hat{\rho} = \frac{1}{4} \sum_{i,j=0}^{3} r_{i,j}\, \hat{\sigma}_i \otimes \hat{\sigma}_j. \tag{12}$$

* Electronic address: ekarimi@uottawa.ca
